# Brain Energy and Oxygen Metabolism: Emerging Role in Normal Function and Disease

**DOI:** 10.3389/fnmol.2018.00216

**Published:** 2018-06-22

**Authors:** Michelle E. Watts, Roger Pocock, Charles Claudianos

**Affiliations:** ^1^Queensland Brain Institute, The University of Queensland, St. Lucia, QLD, Australia; ^2^Development and Stem Cells Program, Department of Anatomy and Developmental Biology, Monash Biomedicine Discovery Institute, Monash University, Clayton, VIC, Australia; ^3^Centre for Mental Health Research, The Australian National University, Canberra, ACT, Australia

**Keywords:** oxidative metabolism, hypoxia, neurometabolism, plasticity, neurodegeneration

## Abstract

Dynamic metabolic changes occurring in neurons are critically important in directing brain plasticity and cognitive function. In other tissue types, disruptions to metabolism and the resultant changes in cellular oxidative state, such as increased reactive oxygen species (ROS) or induction of hypoxia, are associated with cellular stress. In the brain however, where drastic metabolic shifts occur to support physiological processes, subsequent changes to cellular oxidative state and induction of transcriptional sensors of oxidative stress likely play a significant role in regulating physiological neuronal function. Understanding the role of metabolism and metabolically-regulated genes in neuronal function will be critical in elucidating how cognitive functions are disrupted in pathological conditions where neuronal metabolism is affected. Here, we discuss known mechanisms regulating neuronal metabolism as well as the role of hypoxia and oxidative stress during normal and disrupted neuronal function. We also summarize recent studies implicating a role for metabolism in regulating neuronal plasticity as an emerging neuroscience paradigm.

## Introduction

Regulation of tissue metabolite supply and cellular energy metabolism is essential to maintain healthy cellular and systemic function. This regulation is especially critical to the central nervous system (CNS) where energy consumption is highly dynamic. Within the brain, increased neuronal activity drives increased energy consumption and compensatory metabolic and vasculature changes in turn enhance neuronal function (Roy and Sherrington, 1890). Normal brain function therefore requires metabolism to be tightly regulated both temporally and spatially from a regional level down to the level of a single synapse. Currently our knowledge of the relationship between neuronal activity and oxygen metabolism is poorly understood and it is likely that numerous mechanisms and complex regulatory pathways are yet to be uncovered.

While making up only a small fraction of our total body mass, the brain represents the largest source of energy consumption—accounting for over 20% of total oxygen metabolism. Of this, it is estimated that neurons consume 75%–80% of energy produced in the brain (Hyder et al., 2013). This energy is primarily utilized at the synapse with a large proportion spent in restoration of neuronal membrane potentials following depolarization (Harris et al., 2012). Other neuronal functions such as vesicle recycling, neurotransmitter synthesis and axoplasmic transport also contribute to synaptic energy depletion and the requirement for an elevated metabolic rate in neurons (Attwell and Laughlin, 2001; Rangaraju et al., 2014; Pathak et al., 2015). Energy requirements are therefore not uniform throughout the brain but instead increased in localized regions dependent on neuronal activity. While mechanisms have been identified to modify oxygen supply to brain regions in response to activity there appears to be a role for hypoxia in modulating neuronal function and behavior. Disruption of oxygen metabolism and mitochondrial function are also consistent pathological features of various age-related neurodegenerative diseases associated with cognitive decline (Tabrizi et al., 2000; Silverman et al., 2001; Zhou et al., 2008). Despite this, the underlying molecular mechanisms preceding neurodegeneration remain relatively unknown. In recent years a number of studies have identified links between metabolically regulated genes and behavior, which may provide insight into understanding the role of neuronal oxidative metabolism in both health and disease.

## Neurovascular and Neurometabolic Coupling

To compensate for varying energy demands throughout the brain and to increase efficiency of metabolite supply, neurovascular and neurometabolic coupling mechanisms have evolved to enhance blood flow and utilization of metabolites in areas of neural activity.

### Neurovascular Coupling

Cerebral blood flow (CBF), blood volume, glucose consumption and oxygen metabolism are all increased within localized regions of activity following neuronal stimulation. Neurovascular coupling, first postulated by Roy and Sherrington (1890) forms the basis of many functional neuroimaging technologies, where areas of neuronal activity are detected by activity-coupled increases in local CBF. While there has been substantial research on neurovascular coupling since this finding, details of the molecular mechanisms are still being uncovered.

**Figure 1:**
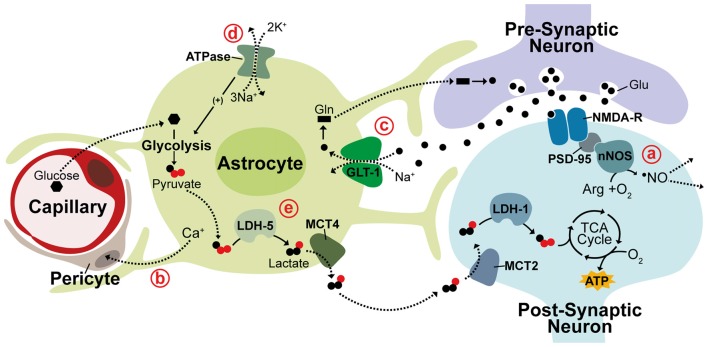
Neurovascular and neurometabolic coupling mechanisms. Schematic illustrating neuronal and astrocytic mechanisms responsible for activity-related blood flow and metabolic changes. **(a)** NMDA receptors (NMDA-R) are linked to neuronal nitric oxide synthase (nNOS) through post-synaptic density protein 95 (PSD-95) and neurovascular coupling during activity is thought to be triggered through the neuronally-produced vasodilator •NO, which can diffuse rapidly and freely through membranes to act on arterioles. **(b)** Vasodilation is also thought to be controlled at the capillary level through astrocytic Ca^+^ signaling acting on contractile perictyes. **(c)** In the glutamine-glutamate cycle, glutamate (Glu) released into the synaptic cleft is cleared by Na^+^-dependent astrocytic uptake, primarily through GLT-1. Glutamate is converted to glutamine (Gln) and returned to neurons to replenish neurotransmitter stores. **(d,e)** The astrocyte-neuron lactate shuttle (ANLS) hypothesis suggests associated increases in astrocytic Na^+^ concentration triggers activation of Na^+^/K^+^ ATPase pumps, promoting glucose uptake and glycolysis. Glycolytically-generated lactate is released and utilized as a substrate for oxidative phosphorylation in neurons during periods of activity. LDH, lactate dehydrogenase; MCT, monocarboxylate transporter. Solid lines indicate enzymatic activity, dashed lines indicate solute movement.

Significant evidence suggests neurovascular coupling is mediated through the free radical, nitric oxide (•NO) produced in neurons. Vasodilation is strongly stimulated by •NO through activation of the enzymatic •NO receptor, soluble guanylate cyclase (sGC), producing cGMP and leading to vasodilation by cGMP-dependent kinase signaling (Miki et al., 1977; Archer et al., 1994). Production of •NO by neuronal nitric oxide synthase (nNOS) is tightly coupled to glutamatergic excitation with activation of nNOS being linked to stimulation of ionotrophic glutamate receptors. This principally occurs through NMDA receptors (NMDA-R) due to strong binding between the NMDA-R clustering protein, post-synaptic density protein 95 (PSD-95), and nNOS (Garthwaite et al., 1988; Brenman et al., 1996). Evidence also suggests that •NO is able to spread rapidly beyond the area of directly activated neurons and is likely to be self-regulating as enhanced blood flow inactivates •NO signaling through increased erythrocyte-mediated scavenging of •NO (Steinert et al., 2008; Santos et al., 2011). Astrocytes also play a role in mediating CBF regulation during neuronal activation by triggering Ca^2+^ release within astrocytic end feet and inducing various downstream Ca^2+^ signaling pathways known to control vasodilation (Mulligan and MacVicar, 2004; Takano et al., 2006). It recently became clear that astrocytic Ca^2+^ signaling acts on contractile perictyes surrounding capillaries and not on arterioles (Mishra et al., 2016). The current view on neurovascular coupling, therefore, is that increased CBF is triggered by astrocytic Ca^2+^ signaling in the capillary bed and by neuronal •NO generated through NMDA-R activation at the arteriolar level (Figures 1a,b; Peppiatt et al., 2006; Mishra et al., 2016).

### Neurometabolic Coupling

This synergistic function of astrocytes and neurons in CBF regulation is mirrored in their inverse yet complimentary metabolic profiles with astrocytes predominantly metabolizing glucose via glycolysis while neurons rely on oxidative metabolism (Kasischke et al., 2004). Astrocytes closely appose both capillary walls and synaptic clefts and are crucial regulators of neurometabolic coupling during neuronal activity. One of the best-characterized roles of astrocytes in neuronal activation is maintaining neurotransmitter stores through the glutamine-glutamate cycle. Glutamate released into the synaptic cleft during excitation is rapidly cleared by astrocytic uptake, primarily through the Na^+^-dependent glutamate transporter GLT-1 (EAAT2), causing attenuation of postsynaptic activation (Figure 1c; Danbolt et al., 1992; Bergles and Jahr, 1997). Cleared glutamate is primarily converted by astrocytes into glutamine, which is then released back into extracellular space for neuronal re-uptake and conversion back to glutamate (Hertz et al., 1978; Kvamme, 1998). In the astrocyte-neuron lactate shuttle (ANLS) hypothesis, proposed by Pellerin and Magistretti (1994), a secondary effect of astrocytic glutamate uptake prompts a switch from oxidative metabolism to aerobic glycolysis in astrocytes causing glucose metabolism to be diverted from the tricaboxcylic acid (TCA) cycle to the glycolytic pathway and lactate production. This switch is thought to be triggered by the associated intracellular increase in Na^+^ concentration, which activates Na^+/^K^+^-ATPase pumps stimulating glucose uptake and glycolysis (Figure 1d; Pellerin and Magistretti, 1997). This adaptation seems to support an increased neuronal metabolic load with lactate generated from astrocytic glycolysis being utilized as a substrate for oxidative metabolism in neurons. This hypothesis is supported by numerous studies detecting increased lactate in regions of brain activity as well as evidence that lactate is crucial for synaptic transmission in rat hippocampal slices and sufficient to support synaptic activity in the absence of glucose (Figure 1e; Schurr et al., 1988, 1999; Frahm et al., 1996; Maddock et al., 2009; Suzuki et al., 2011; Schaller et al., 2014; Machler et al., 2016).

This segregated metabolism is supported by distinct gene expression patterns observed in neurons and astrocytes. Differential expression of lactate transporter proteins, monocarboxylate transporters (MCTs), supports shuttling of lactate from astrocytes to neurons. The lactate efflux transporter MCT4 is expressed primarily in astrocytes while MCT2, an isoform that allows for rapid substrate uptake of lactate, is primarily expressed in neurons (Debernardi et al., 2003; Rafiki et al., 2003). Additionally, the lactate dehydrogenase (LDH) isoenzyme, LDH-5, which promotes conversion of pyruvate to lactate is highly expressed in astrocytes but not in neurons while LDH-1, which promotes pyruvate production is found in both neurons and astrocytes (Bittar et al., 1996; Bröer et al., 1997). In support of glycolysis induction in astrocytes, the pyruvate dehydrogenase kinase-4 (PDK4) is expressed at high levels in astrocytes causing its target, pyruvate dehydrogenase (PDH), to remain in an inactive, phosphorylated state thereby decreasing pyruvate entry into the TCA cycle (Halim et al., 2010; Zhang et al., 2014). Correspondingly, astrocytes express higher levels of the glyoxalase enzymes Glo-1 and Glo-2 that detoxify methyglycoxal, a metabolic by-product of glycolysis (Belanger et al., 2011). An enzymatic promoter of glycolysis, 6-phosphofructo-2-kinase/fructose-2,6-bisphosphate 3 (Pfkfb3), is also found to be functional in astrocytes but subject to constant degradation in neurons contributing to the diversion of neuronal glucose from glycolysis to the pentose-phosphate pathway (PPP; Herrero-Mendez et al., 2009; Belanger et al., 2011; Zhang et al., 2014). While there is substantial evidence in support for the ANLS acting as a mechanism for coupling of neuronal activity to neuronal metabolism, contradictory evidence continues the debate of this hypothesis. Glucose uptake and phosphorylation has been shown to preferentially occur in neurons, not astrocytes. Further, neurons metabolize substantial amounts of glucose and increase glucose metabolism in response to activity (Patel et al., 2014; Lundgaard et al., 2015). This contradictory evidence may be due to metabolism being differentially regulated within different neural networks or brain regions. These observations all contribute, however, to mounting evidence suggesting that neurons can sustain and enhance oxidative metabolism to meet energetic requirements during periods of activity.

## Oxidative Metabolism and Hypoxia

### Oxygen Concentration in the Brain

While there is significant evidence to support enhanced neuronal oxidative metabolism during activity, what remains unclear is what is happens to cellular oxygen concentration following activation. This is partly due to difficulties in recording oxygen concentration as well as from confounds in interpreting oxygen consumption imaging signals. Blood-oxygen-level dependent (BOLD) fMRI which relies on neurovascular coupling to measure regions of brain activity based on measurements of oxyhemeoglobin and deoxyhemeoglobin consistently generates signals with a post-stimulus undershoot (van Zijl et al., 2012). The physiological basis of the BOLD undershoot is heavily debated and is likely stimulus-dependent, one theory however suggests that the BOLD undershoot reflects an uncoupling of CBF and energy metabolism. This is supported by evidence that oxidative metabolism remains elevated post activation after both blood flow and blood volume have returned to baseline (Lu et al., 2004). Consistent with this, numerous studies have reported similar increases in oxidative metabolism indicating that sustained focal activation raises the rate of oxidative metabolism to a new steady state level (Hoge et al., 2005; Mangia et al., 2007; Frahm et al., 2008; Donahue et al., 2009; Lin et al., 2010). With dynamic changes in oxygen metabolism occurring during neuronal activity, dynamic changes are likely to be reflected in levels of oxygen concentration, potentially having secondary effects on protein function and gene expression.

Neurons and neuronal functions are generally viewed as highly sensitive to hypoxia with disruption of oxygen supply to the brain causing detrimental damage within minutes. Although there is not a clearly defined “critical” oxygen tension (PtO_2_) at which hypoxic damage will occur in neurons, in rat cortex a PtO_2_ value between 6.8 mm Hg and 8.8 mm Hg has been estimated as a PtO_2_ where oxidative metabolism will be disrupted (Rolett et al., 2000). Under physiological conditions, PtO_2_ measurements in rat range from 6 mm Hg to 40 mm Hg within the cortex (6–16 mm Hg in white matter and 19–40 mm Hg in gray matter) and from 1 mm Hg to 60 mm Hg across all brain regions with proximal structures displaying large variations in oxygen tension (Erecińska and Silver, 2001). During embryonic development, oxygen tension is low in the fetal brain (0.076–7.6 mm Hg) and hypoxia is essential for proper embryo morphological development. Within the developing brain, oxygen tension acts as a regulator of neurogenesis with low oxygen promoting progenitor expansion in cortical neurogenic regions and decreasing dopaminergic neurogenesis in the midbrain (Wagenführ et al., 2015, 2016). Additionally, in the adult brain, hypoxic injury caused by ischemic stroke triggers increased neuronal stem cell proliferation and neurogenesis (Arvidsson et al., 2002; Macas et al., 2006; Martí-Fàbregas et al., 2010). This evidence supports a role for hypoxia as a regulatory mechanism in neuronal function and indicates that physiological hypoxia occurring in the adult brain may play a functional role.

### Hypoxia Inducible Transcription Factors

**Figure 2:**
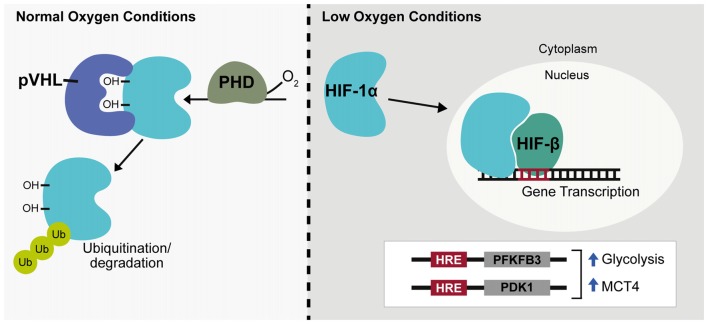
Hypoxia inducible transcription factor regulation. Under normal oxygen conditions hypoxia-inducible factor-1α (HIF-1α) is hydroxylated by prolyl hydroxylase (PHD) enzymes and targeted for ubiquitination by the Von Hippel-Lindau tumor suppresser ubiquitin ligase complex (pVHL). During hypoxia or low oxygen conditions, HIF-1α is stabilized, translocates to the nucleus and associates with HIF-β to promote gene expression, targeting genes containing a hypoxia response element (HRE). HIF-1α acts as a glycolytic enhancer through transcriptional activation of metabolic genes including 6-phosphofructo-2-kinase/fructose-2,6-bisphosphate 3 (PFKFB3) and pyruvate dehydrogenase kinase-1 (PDK1), both positive regulators of glycolysis and monocarboxylate transporter 4 (MCT4), the lactate efflux transporter. Ub, ubiquitin; OH, hydroxyl group.

Long-term changes in cellular response to hypoxia are mediated through changes in gene expression with hypoxia predicted to regulate around 1%–1.5% of the genome, primarily through the hypoxia-inducible factors (HIFs; Koong et al., 2000; Denko et al., 2003). HIF is a heterodimeric complex consisting of a constitutively expressed β subunit shared by a family of three oxygen-sensitive α subunits. Most widely studied among these is the HIF-1α subunit. HIFα protein is constitutively expressed but is immediately targeted for degradation by HIF prolyl hydroxylases (PHDs) that associate with and hydroxylate two conserved HIFα proline residues in an oxygen dependent manner (Bruick and McKnight, 2001). The Von Hippel-Lindau tumor suppressor ubiquitin ligase complex (pVHL), subsequently recognizes HIFα causing HIFα ubiquitination and protein degradation (Ivan et al., 2001; Jaakkola et al., 2001). During hypoxia, though oxygen-limited inactivation of HIF PHD activity, HIFα is no longer targeted by pVHL and is able to accumulate in the cytoplasm before translocating to the nucleus and acting to promote transcription (Figure 2). Within the nervous system HIF-1α and target genes of HIF-1 are widely expressed under hypoxia, but regulation of HIF-1α can differ among neuronal subtypes (Bergeron et al., 1999; Stroka et al., 2001). Following hypoxia, HIF-1α has been shown both *in vitro* and *in vivo* to be significantly upregulated in interneurons but not in pyramidal neurons and in neuronal and non-neuronal cells it has been established that the redox state of a cell contributes to HIF-1α regulation (Welsh et al., 2002; Ramamoorthy and Shi, 2014). Additionally, during in *C. elegans* development, hypoxia has been shown to cause defects in axonal migration that occur in a neuronal cell-type specific manner and are dependent on stabilization of Hif-1 by either hypoxia or increased reactive oxygen species (ROS; Pocock and Hobert, 2008). Being a primary source of reducing agents, glucose is a major contributor to the redox state of a cell and HIF-1α expression in neurons has been shown to increase in a glucose-dependent manner during hypoxia (Shi and Liu, 2006; Guo et al., 2008). There is also a negative relationship between HIF-1α and ROS levels indicating ROS promotes HIF-1α degradation while a reducing environment stabilizes HIF-1α (Schafer and Buettner, 2001; Niecknig et al., 2012).

ROS are highly reactive free radical molecules that can cause cellular damage through oxidation of lipids, proteins and DNA. ROS production primarily occurs through electron leakage at electron transport chain (ETC) complexes I or III during normal oxidative respiration. This causes conversion of 1%–2% of oxygen into the superoxide anion, a precursor to hydrogen peroxide and hydroxyl free radicals. Within the brain, a high neuronal oxidative rate heightens the potential for ROS production and neurons are especially vulnerable to oxidative damage due to low levels of antioxidant enzymes such as glutathione (GSH; Dringen et al., 1999). Neuronal diversion of glucose catabolism from glycolysis to the PPP through Pfkfb3 degradation therefore not only supports oxidative metabolism of lactate but also enhances neuronal antioxidant capacity through production of the reducing agent, NADH. HIF-1α is also involved in this process and acts as a glycolytic enhancer through transcriptional activation of metabolic genes including Pfkfb3 and pyruvate dehydrogenase kinase-1 (PDK1), both positive regulators of glycolysis and the lactate efflux transporter, MCT4 (Figure 2; Minchenko et al., 2002; Kim et al., 2006; Ullah et al., 2006).

As an oxygen-sensitive molecule, which is highly integrated into metabolic processes, HIF-1α is likely to have an important role in brain plasticity, and dysregulation of HIF-1α expression has already been implicated in neuronal activation and learning and memory. In a rat microarray study, seizures induced by injection of Kainate, a potent glutamate-receptor agonist that causes overstimulation of neurons, resulted in a 2.2-fold increase in HIF-1α after 24 h (Hunsberger et al., 2005). In another microarray study HIF-1α was found to be increased 7-fold in mice following environmental enrichment, where mice are exposed to heightened sensory stimulation known to promote neurogenesis and improve performance in memory tasks (Rampon et al., 2000). Elevated HIF-1α levels have also been observed in rats following learning in the Morris water maze and analysis of genes upregulated at early-time points following Morris water maze tests has found an over-representation of HIF-binding sites, hypoxia response elements (HREs), in their promoters (O’Sullivan et al., 2007). These data support a significant role for hypoxia in neuronal activity, potentially though neurovascular uncoupling and enhanced neuronal oxidative metabolism depleting neuronal oxygen levels.

## Disrupted Metabolism in Neurodegenerative Disorders

### Alzheimer’s Disease

Neurodegenerative disorders encompass a range of conditions characterized by progressive neuronal damage and degeneration as well as neuronal cell death. Although neurodegenerative disorders vary in the neuronal populations and cognitive or motor functions affected, metabolic dysfunction is a unifying pathology underlying many of these disorders. The most prevalent and most extensively studied of these is Alzheimer’s disease (AD) occurring in around 1:10 people aged over 65. AD principally affects short-term working memory and is classified by the presence of two hallmark neuropathologies; extracellular amyloid plaques, formed from aggregation of amyloid (Aβ) peptide, and intraneuronal neurofibrillary tangles formed from aggregation of hyperphosphorylated tau. In AD patients, regional hypometabolism in the brain is a predictor for progressive cognitive decline and reduced cerebral metabolism is associated with carriers of the AD risk allele of the APOE-4 gene (Small et al., 1995; Silverman et al., 2001). At the cellular level, mitochondria (MC) isolated from AD patients display reduced enzymatic activity of the ETC complex IV (cytochrome C oxidase; Parker et al., 1990; Parker and Parks, 1995). Similarly, in mouse models of AD, oxidative respiration is diminished and Aβ is found to localize and progressively accumulate in neuronal MC (Mucke et al., 2000; Manczak et al., 2006; Rhein et al., 2009; Yao et al., 2009). This progressive accumulation of Aβ in MC is associated with reduced oxidative respiration and reduced activity of the rate-limiting TCA cycle enzyme, α-ketoglutarate dehydrogenase complex (KGDHC), and the pyruvate dehydrogenase complex (PDHC), which generates acetyl-CoA for entry into the TCA cycle (Casley et al., 2002). Both metabolic dysfunction and mitochondrial Aβ accumulation appear to occur early in disease progression, preceding the onset of extracellular plaque formation (Wirths et al., 2001; Du et al., 2010). This indicates that early metabolic dysfunction is a key process in AD progression and a potential target for therapeutic intervention.

Also preceding extracellular plaque formation in the AD brain significantly increased ROS production and oxidative stress. Substantially increased ROS activity and oxidative damage is consistently detected in AD patients by various measures (Hensley et al., 1995; Gabbita et al., 1998; Praticò et al., 1998; Calingasan et al., 1999; Greilberger et al., 2008). Increased oxidative stress occurs early in disease progression being observed in patients with mild AD as well as in cases of mild cognitive impairment, at high-risk of developing AD (Baldeiras et al., 2008). The pathological Aβ is also known to be a source of ROS production and a cause of neuronal oxidative damage in AD (Behl et al., 1994; Harris et al., 1995; Bianca et al., 1999).

Related to oxidative stress, and also implicated in AD pathology, is dysregulated homeostasis of redox transition metal ions including zinc, copper and iron (Schrag et al., 2011; Ventriglia et al., 2012; Ayton et al., 2015). Both elevation and deficiency of zinc is associated with AD and evidence suggests that altered compartmentalization of zinc rather than altered zinc levels may be the cause of zinc pathology in AD (Suh et al., 2000; Schrag et al., 2011). This is supported by dysregulation of numerous zinc transporters in AD patient brains (Lovell et al., 2005, 2006; Beyer et al., 2009). Zinc has important roles in normal neuronal function and is co-released along with glutamate at the synapse (Vogt et al., 2000). A major role of zinc is its significant antioxidant capacity, such that zinc deficiency is linked to neuronal oxidative stress (Aimo et al., 2010). Like zinc, copper elevation and copper deficiency have both been associated with AD as well as co-localization of copper with Aβ plaques (Miller et al., 2006; Schrag et al., 2011; Ventriglia et al., 2012). Copper is also modulated by synaptic activation in neurons and both zinc and copper are able to bind Aβ (Schlief et al., 2005; Tõugu et al., 2008). In AD pathology, copper enhances Aβ toxicity and copper:Aβ complexes are a source of ROS production and oxidative damage in neurons (Dikalov et al., 2004; Liu et al., 2008; Ellis et al., 2010).

The redox active iron, although vital for cellular function, is also a pro-oxidant and promotes generation of highly reactive hydroxyl radicals from hydrogen peroxide. Elevated levels of brain iron in the AD brain as well as iron association with Aβ plaques and neurofibrillary tangles have been detected in various studies (Smith et al., 1997; Bartzokis et al., 2000; Raven et al., 2013). Recently, elevated iron has been shown to predict AD progression and elevated iron was linked to the APOE-4 AD risk allele suggesting it may have a pathological role in AD (Ayton et al., 2015).

**Figure 3:**
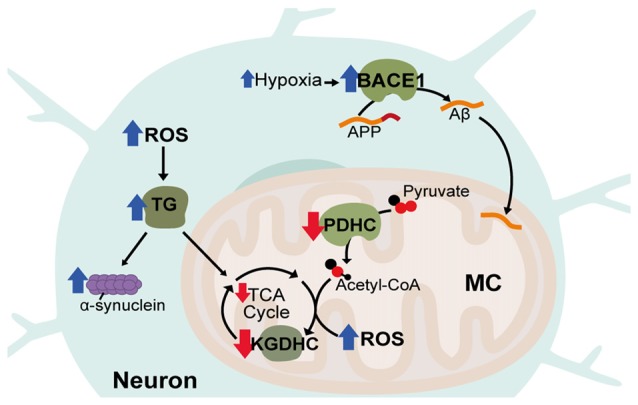
Disrupted metabolic pathways in neurodegenerative diseases. Hypoxia associated with Alzheimer’s Disease (AD) leads to increases in the HIF-1α target, β-site β-amyloid precursor protein cleavage enzyme 1 (BACE1), which cleaves amyloid precursor protein (APP) to produce Aβ. Aβ accumulates in neuronal mitochondria (MC) early in disease progression and disrupts oxidative metabolism. Acetyl-CoA production and tricaboxcylic acid (TCA) cycle entry is decreased in AD through reduced activity of the pyruvate dehydrogenase complex (PDHC). In all three diseases, activity of α-ketoglutarate dehydrogenase complex (KGDHC) is reduced, reactive oxygen species (ROS) is increased and transglutaminase (TG) activity is increased. TG increases α-synuclein aggregation and reduces oxidative respiration.

Another common feature of AD that contributes to AD pathology is vascular dysfunction. Cerebrovascular disease, characterized by disrupted blood flow to the brain, significantly increases AD risk and occurs before Aβ accumulation and cognitive decline (Arvanitakis et al., 2016). In animal models, hypoperfusion also leads to symptoms similar to AD and exacerbates existing AD pathology (Walsh et al., 2002; Wang et al., 2010b). Vascular dysfunction contributes to the pathology of AD due to lower capillary density, meaning narrowed blood vessels and decreased CBF (Hamel et al., 2008). Diminished blood flow reduces metabolite and oxygen supply to the brain and potentially contributes to build-up of Aβ through impaired clearance of neurotoxic molecules (Shibata et al., 2000; Kumar-Singh et al., 2005). Aβ itself is also thought to amplify deficits in CBF and glucose utilization in AD through impairing vasodilation and cerebrovascular autoregulatory mechanisms (Niwa et al., 2002). Cerebrovascular dysfunction can lead to disrupted oxygen metabolism through hypoperfusion-hypoxia and hypoxia in-turn can enhance AD pathology by promoting tau phosphorylation as well as transcriptionally upregulating the HIF-1 target, β-site β-amyloid precursor protein cleavage enzyme 1 (BACE1) that cleaves amyloid precursor protein (APP) to produce Aβ (Figure 3; Sun et al., 2006).

### Parkinson’s and Huntington’s Disease

Aside from rare cases of genetic mutations in familial AD, the major risk factor for developing AD is aging. Correspondingly, AD, shares a number of similarities with other late-onset neurodegenerative disorders including Parkinson’s Disease (PD) and Huntington’s disease (HD). PD is thought to be caused by both genetic and environmental factors and primarily impacts patient motor function. PD involves the formation of protein aggregates consisting mainly of α-synuclein and affects the dopaminergic neurons of the midbrain substantia nigra. HD is an inherited neurodegenerative disorder caused by expanded CAG repeats in the Huntingtin (HTT) gene causing progressive neuronal degeneration and cell death throughout the brain, affecting mood, cognition and motor skills. Inclusions are also found in the HD brain from aggregation of mutant HTT (mHTT) protein. Like AD, both PD and HD are associated with increased oxidative stress as well as decreased activity of the KGDHC enzyme (Tabrizi et al., 2000; Gibson et al., 2003; Klivenyi et al., 2004; Zhou et al., 2008). Also, common to all three disorders is increased activity of transglutaminase (TG; Johnson et al., 1997; Junn et al., 2003; Jeitner et al., 2008). TG catalyzes polyamination post-translational modifications of proteins, is known to be increased by ROS and also attenuates HIF-1 signaling (Campisi et al., 2004; Filiano et al., 2008). TG can decrease oxidative metabolism through modification of glycolytic enzymes and is known to cause oxidative stress in HD and aggregation of α-synuclein in PD (Cooper et al., 1997; Junn et al., 2003; Kim et al., 2005).

Mutations in mitochondrial genes have also been identified in cases of familial PD and exposure to the neurotoxin MPP+, which inhibits ETC Complex I and therefore oxidative respiration, causes permanent Parkinsonism (Langston et al., 1983; Parker and Parks, 2005; Plun-Favreau et al., 2007). Altered metal ion homeostasis may have a role in PD pathology as well with disrupted levels of both zinc and copper observed in PD patients (Brewer et al., 2010; Davies et al., 2014). Similar to Aβ, copper also contributes to α-synuclein aggregation and can contribute to oxidative stress through the formation of reactive copper: α-synuclein complexes (Wang et al., 2010a; Dell’Acqua et al., 2015). α-synuclein is also know to exacerbate mitochondrial dysfunction in the presence of toxic oxidizing agents, with loss of α-synuclein in animal models conferring resistance to mitochondrial toxins (Klivenyi et al., 2006; Norris et al., 2007). Additionally, levels of α-synuclein are increased when oxidative metabolism is inhibited and animal models expressing mutant forms of α-synuclein exhibit neuronal mitochondrial degeneration and cell death (Lee et al., 2002; Martin et al., 2006). In HD, increased oxidative damage to mitochondrial DNA is observed as well as higher frequencies of deletions in the mitochondrial genome and deficits in ETC function with decreased expression of complex II in the striatum and decreased activity of complex IV in striatal and cortical regions (Horton et al., 1995; Polidori et al., 1999). Neuronal mitochondrial permeability is also disrupted by the mHTT protein through increasing sensitivity of the permeability transition pore to Ca^2+^ concentration, leading to mitochondrial dysfunction and decreased ATP production (Brustovetsky et al., 2003; Milakovic et al., 2006). Vascular deficits and disrupted blood flow is a major pathology of HD as well with altered blood vessel density and size found in cortical gray matter, putamen and striatal brain regions. In HD patients, inclusions of mHTT are also detected in the basal membrane and epithelium of cortical blood vessels and in mouse models of the disease pericytic coverage of cortical and striatal blood vessels is decreased (Drouin-Ouellet et al., 2015; Hsiao et al., 2015).

### Aging

A number of the metabolic pathologies observed in neurodegenerative disorders are associated with normal aging and may explain the age-related manifestation of neurodegenerative disease phenotypes. While no longer thought to be directly causative of aging, free radicals and oxidative stress accumulate in the aging brain as in neurodegeneration (Smith et al., 1992). Mitochondrial function is also linked to aging due to the association of mitochondrial DNA (mtDNA) haplotypes with longevity and the generation of mtDNA mutator mice that have a premature aging phenotype (Trifunovic et al., 2004; Alexe et al., 2007; Bilal et al., 2008). It has also been shown there is an increased rate of damaging mutations in mtDNA of post-mitotic aging cells as opposed to aging mitotic cells (Greaves et al., 2012). While it has been suggested that the somatic rate of mtDNA mutation is unlikely to have a pathological affect due to redundancy in cell mitochondrial numbers, in post-mitotic neurons mtDNA mutation rates are significantly higher than average and, within the cortex, MC with large mtDNA deletions possess a replicative advantage during mitochondrial expansion (Song et al., 2005; Bender et al., 2006; Kraytsberg et al., 2006; Fukui and Moraes, 2009). Aside from AD and PD, deficiency of zinc is also associated with aging, being decreased in the general elderly population (Pepersack et al., 2001). Diminished CBF occurs in normal aging as well with cortical perfusion found to decrease with age in healthy adults (Chen et al., 2011). An age-dependent reduction in perictyes also occurs in mice and is associated with microvascular changes and neurodegeneration (Bell et al., 2010). Substantial evidence therefore exists supporting disrupted neuronal oxygen supply and oxidative metabolism as a major pathological component of age-related neurodegeneration.

## Oxygen Metabolism as a Driver of Neuronal Plasticity

Although it has been well established that metabolic regulation is critical to neuronal function and that metabolic dysfunction is a major pathology in diseases affecting behavior and cognition, there is little known regarding how regulators of metabolism may be involved in neuronal plasticity. A number of studies, however, support a direct role for metabolic regulation and metabolically linked genes in influencing learning and memory. One of the best examples of this is exposure of hypoxia as a modulator of cognitive performance. In *C. elegans*, hypoxia acts as an enhancer of gustatory sensory perception through Hif-1 dependent induction of the neurotransmitter serotonin within specific sensory neurons (Pocock and Hobert, 2010). In rodent models, exposure to hypobaric hypoxia in adult rats for periods of 7–21 days causes decline in spatial learning similar to aging and is associated with aging-related lipofuscin deposition and ultrastructural changes in MC. Increasing duration of hypobaric hypoxic exposure also positively correlates with increasing expression of aging markers (Biswal et al., 2016). Brief hypoxic exposure (100 s) in rats also causes synaptic arrest of pyramidal CA1 hippocampal neurons and deficits in spatial memory that are both reversed by blockade of receptors for Adenosine, an inhibitory neurotransmitter (Sun et al., 2002). Intermittent hypoxia (90–120 s intervals of 6%–10% O_2_ for 10 h/day) also produces deficits in acquisition of spatial memory in adult rats that could be prevented by administration of antioxidant (Row et al., 2003; Ward et al., 2009). In contrast, long-term facilitation of motor output in adult rats is enhanced by intermittent hypoxia (3 × 3 min intervals, separated by 5 min hyperoxia) increasing both phrenic amplitude and burst frequency, which was not observed with a continuous hypoxia of the same cumulative duration (Baker and Mitchell, 2000). Differing effects of hypoxia in brain plasticity are likely related to differing exposures as well as measurement of different outputs. Interestingly, mild hypoxia preconditioning confers protection of cognitive abilities during subsequent exposure to severe hypoxia implicating a role for HIFs and transcriptional changes induced by mild hypoxia (Rybnikova et al., 2005). Indeed, neuronal knockout of HIF-1α in mice impairs spatial memory and the stabilization of HIF improves hippocampal memory in fear conditioning (Tomita et al., 2003; Adamcio et al., 2010). Similar learning deficits and age-related changes are also observed in a D-galactose induced model of aging where oxidative injury was the major stimulus for aging (Li et al., 2016).

In learning and memory studies using an inhibitory avoidance paradigm, changes in metabolic gene expression were observed at 24 h, with increased expression of Na^+^/K^+^ ATPase, Glut1, Glut3 and, most prominently, lactate transporters MCT1 and MCT4 detected, suggesting transcriptional modulation of neurometabolic coupling occurs following learning (Yao et al., 2009; Tadi et al., 2015). Altered expression of lactate metabolic enzymes and transporters is also related to stress induced improvements in cognitive function. Psychological stress, while harmful under chronic conditions, has evolved to enhance cognitive function and improve reactions to stressful situations through hypothalamic activation of adrenergic receptors and hypothalamic-pituitary-adrenal axis glucocorticoid production (Dong et al., 2017). In a mouse model of stress, induced by activation of the β2 adrenergic receptor (β2AR), cognitive function was improved with short-term (3–5 days) activation while longer activation (>6 days) was harmful. Improved cognitive function following short-term stress induction corresponds with β2AR-dependant increases in LDH A, MCT1 and MCT4 expression, the expression of which was modulated by β-arrestin-1 activation of HIF-1α, downstream of β2AR (Dong et al., 2017).

Altered expression of ETC oxidative phosphorylation genes is also associated with altered behavior in the honeybee. In a study exploring molecular profiles in aggressive honeybee behavior, oxidative phosphorylation was most significantly enriched in association with increased aggression. This was found to be true for aged bees that display increased aggressive behavior as well as following environmentally enhanced aggression by alarm pheromone exposure and genetic-related aggression occurring in the Africanized honeybee population (Alaux et al., 2009). Consistent with this, inhibition of oxidative phosphorylation by treatment with drugs targeting the TCA cycle increased aggression of honeybees measured using an intruder assay (Li-Byarlay et al., 2014). In the same study, cell-type-specific knockdown of ETC complex genes using GAL4 drivers in *Drosophila* found that neuron-specific, but not glia-specific knockdown of the complex I gene ND20-like, significantly increased aggressive lunging behavior in flies (Li-Byarlay et al., 2014).

Also involved in learning and memory are non-coding miRNA genes which are regulated during neuronal activity by various mechanisms and able to regulate translation of various downstream target genes. A number of miRNAs have been associated with plasticity including the hypoxia-regulated, HIF-1 target, miR-210 that is known to be involved in metabolic regulation. miR-210 is significantly upregulated 24 h after long-term memory formation in the honeybee using an olfactory conditioning paradigm. Upregulation of miR-210 correlated with downregulation of a number of metabolically linked protein-coding genes including Gapdh2, Glucose dehydrogenase, Laccase2 and Aldose reductase-like. Inhibition of miR-210 by treatment of honeybees with miR-210 antogmiR also resulted in reduced memory retention in the olfactory conditioning assay indicating a functional role in learning and memory (Cristino et al., 2014). Considering the sensitivity of neurons and neural structures to hypoxia, Cristino et al. (2014) suggest small changes to oxygen levels in metabolic activity neurons may induce expression of miR-210, which in turn targets key molecules, including plasticity molecules, asparagine synthetase (involved in the biosynthesis of Glutamate) and actin. A follow-up study found that in a human-derived neuronal cell-line, miR-210 targeted neurodegeneration-associated genes as well as other plasticity-related genes within the human transcriptome. This included a number of oxidative metabolism genes, the AD risk-gene *APOE* as well as the NMDA-R, *GRINA*, and the human actin homolog, *ACTB* (Watts et al., 2018). Another hypoxia-regulated miRNA, miR-181c, is also associated with modulating cognitive function in rats. In a model of chronic cerebral hypoperfusion miR-181c was continuously inhibited, correlating with upregulation of its plasticity-related target gene, TRIM2. Hypoperfusion in this model was associated with deficits in spatial learning that were ameliorated by hippocampal overexpression of miR-181c (Fang et al., 2017). These studies all provide support to the hypothesis that metabolically regulated genes are directly involved in the regulation of neuronal plasticity.

## Conclusion

While neurovascular coupling mechanisms appear to maintain steady-state oxygen levels in the brain, it is becoming evident that neurovascular uncoupling may in fact have a physiological role in regulating plasticity via oxygen depletion and induction of downstream hypoxia response pathways. Disruptions to hypoxia and oxidative metabolism have also been extensively attributed to neurodegeneration pathology albeit, there is a lack of understanding, as to how these disruptions are triggered and how they may be therapeutically targeted to halt disease progression and improve cognitive and motor functions. Altered behavior, including learning and memory, associated with dysregulation of metabolic genes highlights the importance of understanding the role of oxygen metabolism in neuronal plasticity. Further elucidation of how the hypoxia response pathway and other metabolic genes are involved in neuronal function will be critical in determining the molecular links between cognitive function and oxidative metabolism. This in turn will help elucidate how disrupted metabolism can lead to cognitive deficits and neurodegenerative disease.

## Author Contributions

MW wrote the manuscript. RP and CC edited the manuscript.

## Conflict of Interest Statement

The authors declare that the research was conducted in the absence of any commercial or financial relationships that could be construed as a potential conflict of interest.
